# Inhibitory Effect of Valencene on the Development of Atopic Dermatitis-Like Skin Lesions in NC/Nga Mice

**DOI:** 10.1155/2016/9370893

**Published:** 2016-08-18

**Authors:** In Jun Yang, Dong-Ung Lee, Heung Mook Shin

**Affiliations:** ^1^Department of Physiology, College of Korean Medicine Dongguk University, Gyeongju 780-714, Republic of Korea; ^2^Division of Bioscience, Dongguk University, Gyeongju 780-714, Republic of Korea; ^3^National Development Institute of Korean Medicine, Gyeongsan, Gyeongbuk 712-210, Republic of Korea

## Abstract

Valencene (VAL) isolated from* Cyperus rotundus *possesses various biological effects such as antiallergic and antimelanogenesis activity. We investigated the effect of VAL on atopic dermatitis (AD) skin lesions and their molecular mechanisms. We topically applied VAL to 1-chloro-2,4-dinitrobenzene (DNCB) sensitized NC/Nga mice. Modified scoring atopic dermatitis index, scratching behavior, and histological/immunohistochemical staining were used to monitor disease severity. RT-PCR, western blotting, and enzyme-linked immunosorbent assay were used to determine the level of IgE, proinflammatory cytokines/chemokines production, and skin barrier proteins expression. Topical application of VAL significantly reduced AD-like symptoms and recovered decreased expression of filaggrin in DNCB-sensitized NC/Nga mice. The levels of serum IgE, IL-1*β*, IL-6, and IL-13 in skin/splenic tissue were reduced.* In vitro* studies using TNF-*α* and IFN-*γ* treated HaCaT cells revealed that VAL inhibited the exaggerated expression of Th2 chemokines including TARC/CCL17, MDC/CCL22, and proinflammatory chemokines such as CXCL8, GM-CSF, and I-CAM through blockade of the NF-*κ*B pathway. In addition, expression of the skin barrier protein, involucrin, was also increased by VAL treatment. VAL inhibited the production and expression of proinflammatory cytokines IL-1*β* and IL-6 in LPS-stimulated RAW 264.7 cells. These results suggest that VAL may serve as a potential therapeutic option for AD.

## 1. Introduction 

Atopic dermatitis (AD) is a chronic inflammatory, relapsing skin disorder with a high incidence that is frequently associated with elevated production of immunoglobulin E (IgE) and secretion of T helper (Th) 2 cytokines [1]. The diagnosis of AD is based on the following clinical phenotype: dry and eczematous skin, erythematous papules, and severe pruritus [2, 3]. Microscopically, lesional samples of AD patients also show epidermal hyperplasia, acanthosis, and accumulation of lymphocytes and mast cells [4, 5]. Patients with AD generally suffer from severe itch, and the strong action of scratching elicits inflammation of the skin lesions, leading to more itching and exacerbating clinical signs [6, 7]. Therefore, reducing skin inflammation is considered an effective strategy that can prevent aggravation of skin lesions and improve the quality of life for patients with AD.

The etiology of atopic dermatitis is not completely understood, but there have been many studies showing possible mechanisms of AD. Various susceptibility genes, environmental factors, defects in skin barrier, and immune responses play a leading role in AD. Recently, the relationship between skin barrier abnormalities and immune dysregulation has been considered important for the pathogenesis of AD. Epidermal keratinocytes, which form a functional skin barrier, provide the first line of defense against pathogen invasion, irritants, and allergens. In AD patients, allergic sensitization occurs through a damaged skin barrier and leads to immune responses in keratinocytes. Activated keratinocytes in the epidermal lesions of AD are capable of producing thymus and activation-regulated chemokine (TARC)/CCL17 and macrophage-derived chemokine (MDC)/CCL22, which play important roles in the recruitment of Th2 cells into inflammatory skin lesions. Th2 cells induce secretion of IL-4, IL-5, and IL-13, as well as IgE class switching in B cells, which accounts for enhanced IgE levels. Enhanced expression of Th2 cytokines increases epidermal thickening, inflammation, and pruritus, while it downregulates skin barrier proteins including filaggrin, loricrin, and involucrin. Activated keratinocytes also produce various chemokines such as granulocyte-macrophage colony-stimulating factor (GM-CSF), monocyte chemoattractant protein-1 (MCP-1), and C-X-C motif chemokine ligand 8 (CXCL-8), which contribute to the chronicity of inflammation through the infiltration of immune cells.

While acute AD is characterized by infiltration of Th2 cells into skin lesions, chronic AD results in the infiltration of inflammatory dendritic epidermal cells, macrophages, and eosinophils. In particular, macrophages are the source of many cytokines that play fundamental roles in pathogenesis of chronic AD. Activated macrophages have a high capacity to produce inflammatory cytokines such as IL-1*β*, IL-6, IL-12, IL-23, and TNF-*α*, as well as high levels of inducible nitric oxide synthase (iNOS). Activated macrophages also induce neovascularization and contribute to angiogenesis and lymphangiogenesis, which are features of chronic inflammatory disorders.

Nuclear factor-*κ*B (NF-*κ*B) and signal transducer and activator of transcription 1 (STAT1) are important transcription factors associated with the allergic inflammatory response in AD. Upon stimulation, NF-*κ*B and STAT1 in the cytoplasm translocate into the nucleus, where they participate in the expression of many proinflammatory genes. Because inhibition of NF-*κ*B and STAT1 translocation subsequently decrease secretion of inflammatory mediators, they are important pharmacological targets for discovery of novel therapeutics to treat AD.

*Cyperus rotundus *L. is a medicinal plant that has long been used for management of various diseases, including gastric ailments, cognition disorders, wounds, and inflammation [8–12]. Valencene (VAL) is a member of the sesquiterpene and the most well-known bioactive compound isolated from the rhizomes of* C. rotundus* [13]. Previous studies have shown that VAL possesses a wide spectrum of pharmacological properties such as antiseptic, antioxidant, and antiallergic activities [13–15]. Therefore, this compound has the potential to treat inflammatory skin disorders such as AD. However, VAL has not yet been investigated to determine if it suppresses the development of AD. Therefore, the present study was conducted to examine the inhibitory effects of VAL on AD-like lesions in NC/Nga mice, a murine model of AD. Specifically, we assessed clinical symptoms by skin lesion biopsy, spleen tissue, and serum analyses after mice were sacrificed. We confirmed the antiatopic effect of valencene on expression of proinflammatory chemokines and cytokines in HaCaT cells and RAW 264.7 cells* in vitro*. Further, we generated evidence of the underlying mechanisms of these effects and evaluated the action of valencene on skin barrier functions by measuring its effects on expression of skin barrier proteins, such as filaggrin, loricrin, and involucrin.

## 2. Materials and Methods

### 2.1. Plant Materials and Compounds Identification

**Figure 1:**
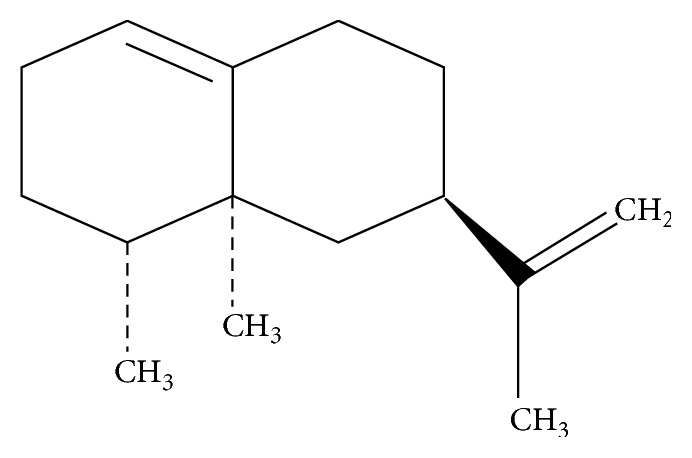
Chemical structure of valencene isolated from the rhizomes of* Cyperus rotundus*.

The rhizomes of* C. rotundus* were purchased from an oriental medicines market (Kyung Dong, Seoul, Korea) and identified by Professor Je-Hyun Lee, College of Oriental Medicine, Dongguk University, Gyeongju, Korea. A voucher specimen (DKH-02561) was deposited at the Ministry of Food and Drug Safety, Korea. The dried rhizomes of this herb (3.0 kg) were chopped and extracted twice with methanol (10 L) under reflux for 3 h to yield the crude extract (342.5 g). A portion (282 g) of this extract was dissolved in distilled water (2.7 L) and partitioned with hexane, yielding the corresponding fractions of 34.61 g, which were subfractionated on a silica gel column by a stepwise elution procedure using hexane-dichloromethane (3 : 1 and 1 : 1), dichloromethane, and hexane-dichloromethane-methanol (10 : 10 : 1) to give seven subfractions (S-1 to S-7). Subfraction S-1 was further chromatographed on a silica gel column using* n*-hexane and* n*-hexane-ethylacetate gradient (100 : 1 to 3 : 1) to give valencene (Figure 1) (1.03 g), which was identified based on comparison of the NMR spectral data with published values [14]. The purity of valencene was determined to be 95.6% by gas chromatography-mass spectrometry (GC-MS) analysis.

### 2.2. Cell Culture

HaCaT cells (a human keratinocyte cell line) and RAW 264.7 cells (a mouse macrophage cell line) were cultured in high glucose Dulbecco's modified Eagle's medium (Gibco Laboratories, Grand Island, NY, USA) containing 10% heat-inactivated fetal bovine serum, 100 units/mL penicillin, and 100 *μ*g/mL streptomycin (Invitrogen, Carlsbad, CA) in a humidified atmosphere of 5% CO_2_ at 37°C. The medium was changed every 2 days during incubation, and cells were made quiescent by starvation in serum-free medium for 24 hours before treatment with the indicated reagents.

### 2.3. Animals and Treatment

NC/Nga (6-week-old, male) mice were purchased from Central Lab Animal Inc. (Seoul, Korea). All animal experimental procedures were performed in accordance with protocols approved by the Institutional Animal Care and Use Committee of Dongguk University. The mice were randomly divided into five groups (*n* = 4): a naïve control group (CON), 2,4-dinitrochlorobenzene (DNCB) group, dexamethasone (DEX) group, low dose of VAL (VAL50) group, and high dose of VAL (VAL100) group. The animals were allowed to acclimate for 1 week prior to any experimental procedures and had their backs shaved. To induce AD-like skin inflammation, the mice in the DNCB, DEX, VAL50, and VAL100 groups received 200 *μ*L of 0.4% 2,4-dinitrochlorobenzene (DNCB, Sigma-Aldrich, St. Louis, MO, USA) dissolved in an acetone and olive oil solution (3 : 1) on shaved dorsal skin twice each week for 5 weeks. After induction of AD, mice in the VAL50, VAL100, and DEX groups received topical application of 200 *μ*L of 50 *μ*M VAL, 100 *μ*M VAL, or 0.1% DEX, respectively, three times a week for 10 weeks. Mice in the CON group received topical application of acetone and olive oil vehicle on their shaved backs. After 10 weeks of treatment, clinical skin severity score and scratching frequency were assessed. Clinical skin scores were calculated using the modified scoring atopic dermatitis (SCORAD) index. A score of 0–3 (0, none; 1, mild; 2, moderate; 3, severe) was assigned for each of four symptoms (erythema/hemorrhage, scarring/dryness, excoriation/erosion, and edema). The frequency of scratching around the dorsal skin lesions with hind paws was counted during a 20-minute period. Under anesthetic conditions, whole blood was collected via cardiac puncture and serum was obtained by centrifugation of the blood (3000 g, 15 min, and 4°C). After sacrifice, the body weight of each mice was recorded. Splenic tissue was excised after elimination of the surrounding connective tissues, rinsed with cold PBS, and then weighed. The lesional skin area of their backs was immediately excised and snap frozen in liquid nitrogen. Skin thickness was determined by measuring the folded dorsal skin with a dial micrometer (Mitutoyo Corporation, Tokyo, Japan).

### 2.4. Histological and Immunohistochemical (IHC) Observation

For histopathological observation, a portion of the skin samples from the back in each group was fixed in 10% formalin for 24 h at 4°C. After paraffin embedding, sections were cut and stained with hematoxylin and eosin (H/E) or toluidine blue (TB) for detection of infiltrated inflammatory cells or mast cells, respectively. For immunohistochemical staining, sections were incubated overnight at 4°C with the indicated primary antibodies. After sections were washed, they were incubated with horseradish peroxidase- (HRP-) conjugated anti-rabbit antibodies or HRP-conjugated anti-mouse antibodies for 1 h at room temperature. Peroxidase activity was visualized with an AEC chromogen kit (Sigma-Aldrich, St. Louis, MO, USA) and examined using a digital camera (Olympus UC30, Japan) mounted on a phase contrast microscope (Olympus CK40-32PH, Japan) using DIXI image solution 2.89 software (DIXI Optics, Daejeon, South Korea).

### 2.5. Enzyme-Linked Immunosorbent Assay (ELISA)

20 mg of splenic or skin tissues was homogenized with ice-cold tissue extraction reagent (Pierce/Thermo Scientific, Rockford, IL, USA), centrifuged at 12,000 g for 20-min, and the supernatant was then separated. The amounts of IL-1*β*, IL-4, IL-6, IL-13, and TNF-*α* in splenic and skin tissues were determined using commercial kits (R&D Systems, Minneapolis, MN, USA) according to the manufacturer's protocol and quantified using standard curves made from serial dilutions of each protein. The serum levels of mouse IgE were measured using commercially available ELISA kits (Koma Biotech, Seoul, Korea). For* in vitro* experiments, HaCaT cells and RAW 264.7 cells were preincubated with VAL for 1 h and then stimulated with TNF-*α* (10 ng/mL)/IFN-*γ* (10 ng/mL) or LPS (1 *μ*g/mL) for 24 h, respectively. The levels of mouse IL-1*β*, IL-6, and TNF-*α* and human CCL17/TARC, CCL22/MDC, CXCL8, MCP-1, GM-CSF, and I-CAM in culture media were determined using commercial kits (Koma Biotech, Seoul, Korea) according to the manufacturer's protocols. Absorbance was measured at 450–550 nm using an automated microplate reader (Molecular Devices, Sunnyvale, CA, USA).

### 2.6. Cell Viability

To investigate the cell toxicity of VAL on HaCaT and RAW 264.7 cells, XTT assay was performed. After treatment of HaCaT and RAW 264.7 cells with VAL for 24 h, 50 *μ*L of XTT solution (Sigma-Aldrich, St. Louis, MO, USA) was added, after which the samples were incubated for additional 4 h. The absorbance was then measured at 450 nm (reference wavelength at 670 nm) using a microplate reader (Molecular Devices, Sunnyvale, CA).

### 2.7. Western Blot Analysis

Collected cells were homogenized in RIPA lysis buffer (Atto, Tokyo, Japan) and then prepared by centrifuging at 10,000 g for 10 min. For nuclear and cytosolic fractions, cells were lysed with nuclear or cytoplasmic extraction reagents (Pierce/Thermo Scientific, Rockford, IL, USA) according to the manufacturer's protocol. Next, 30 *μ*g of proteins was separated by 10–12% SDS-PAGE and transferred onto polyvinylidene difluoride (PVDF) membranes. After blocking for 1 h at 37°C in 5% skim milk (in 1x TBS), the membranes were incubated with primary antibodies and then incubated with horseradish peroxidase-conjugated anti-IgG secondary antibody. Anti-I*κ*B-*α*, P-I*κ*B-*α*, NF-*κ*B p65, STAT1, and P-STAT1 were purchased from Cell Signaling Technology Inc. (Danvers, MA, USA). Antiinvolucrin, loricrin, and filaggrin were purchased from Abcam Inc. (Cambridge, MA, USA). An anti-*β*-actin antibody was purchased from Sigma-Aldrich Inc. (St. Louis, MO, USA). All bands were detected by enhanced chemiluminescence (Bio-Rad, Hercules, CA, USA). The band intensities of specific proteins were quantified using Gelquant 2.7 (DNR Bio-Imaging Systems, Jerusalem, Israel).

### 2.8. Reverse Transcription-Polymerase Chain Reaction (RT-PCR)

Total RNA was collected using TRI Reagent (Fermentas, MD, USA). RNA was isolated, after which cDNA synthesis was performed using a commercial kit (Fermentas, Glen Burnie, MD, USA). The synthesized cDNA was amplified by PCR using an EmeraldAmp PCR Master Mix (Takara Bio Inc., Shiga, Japan) with 1 *μ*M of each primer. The sequences of the RT-PCR primers used were mouse IL-1*β* (sense primer: 5′-AGGACACGACTGCTTTCTTC-3′, antisense primer: 5′-GCACCGCAGTAGGGAAGTGT-3′), mouse IL-6 (sense primer: 5′-GGCTTTTAAGTGGGGCTGTC-3′, antisense primer: 5′-CCCAAGATCCACTGCAAATG-3′), mouse TNF-*α* (sense primer: 5′-GAGAGGAACACGTTCTGGCTCC-3′, antisense primer: 5′-TGCTGGAGGCTGAGGCATCC-3′), mouse GAPDH (sense primer: 5′-CCATGGAGAAGGCTGGGG-3′, antisense primer: 5′-CAAAGTTGTCATGGATGACC-3′), human CCL17/TARC (sense primer: 5′-ATGGCCCCACTGAAGATGCT-3′, antisense primer: 5′-TGAACACCAACGGTGGAGGT-3′), human CCL22/MDC (sense primer: 5′-TCTGCATTCCCTGATCTCCA-3′, antisense primer: 5′-ATTCATGAAGGGAAGTGGGC-3′), human CXCL8 (sense primer: 5′-GAAAACTGGGTGCAGAGGGT-3′, antisense primer: 5′-TCGGATATTCTCTTGGCCCT-3′), human MCP-1 (sense primer: 5′-CACCTTCATTCCCCAAGGGC-3′, antisense primer: 5′-GCTTGTCCAGGTGGTCCATG-3′), human GM-CSF (sense primer: 5′-CAGCCTCACCAAGCTCAAGG-3′, antisense primer: 5′-TCATGAGAGAGCAGCTCCCC-3′), human I-CAM (sense primer: 5′-AGCGTTCTGATCCGGACTCA-3′, antisense primer: 5′-TCAGATTCTGCTGCCTCCGA-3′), human involucrin (sense primer: 5′-TGGAACAGCAGGAAAAGCAC-3′, antisense primer: 5′-ACCTAGCGGACCCGAAATAA-3′), human filaggrin (sense primer: 5′-TCAAGCAGAAGAGGAAGGCA-3′, antisense primer: 5′-AAGCTTCATGGTGATGCGAC-3′), and human loricrin (sense primer: 5′-GCTCTCATGATGCTACCCGA-3′, antisense primer: 5′-CACTGGGGTTGGGAGGTAGT-3′). The RT-PCR reaction was conducted over 30 cycles of 95°C for 10 s (denaturation), 57.5°C for 30 s (annealing), and 72°C for 1 min (elongation). PCR products were electrophoresed on 1% agarose gel at 100 V and photographed using an ultraviolet transilluminator and a digital capture system (DNR Bio-Imaging Systems, Jerusalem, Israel). The band intensities of specific genes were quantified using the Gelquant 2.7 software (DNR Bio-Imaging Systems).

### 2.9. Statistical Analysis

All data were expressed as the means ± SDs of experiments (*n* = 3 per experiment) and analyzed by one-way ANOVA followed by Duncan's multiple range test using the GraphPad Prism 4.0 software (GraphPad Software, San Diego, CA, USA). A* p* value < 0.05 was considered to indicate statistical significance for all samples.

## 3. Results

### 3.1. Effects of VAL on the AD-Like Symptoms in DNCB-Sensitized NC/Nga Mice

**Figure 2:**
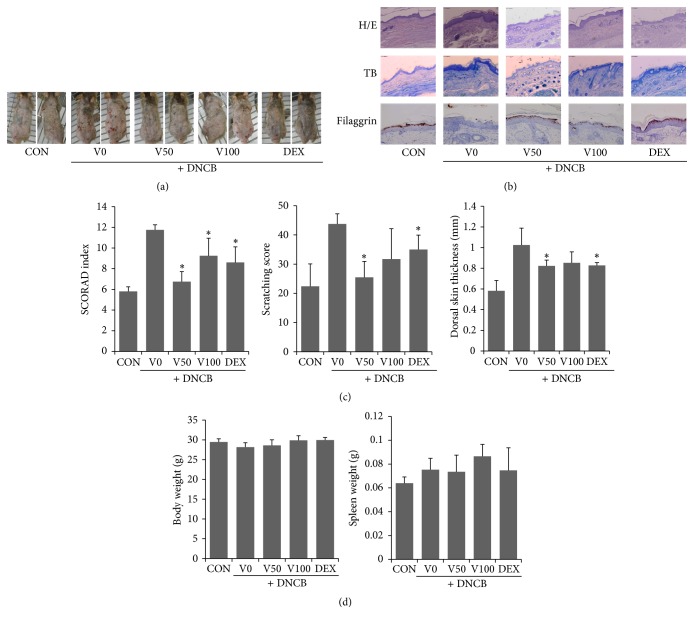
Effects of valencene on DNCB-induced atopy-like dermatitis in NC/Nga mice. (a) Representative clinical features and (b) corresponding histological analyses (hematoxylin and eosin or toluidine blue staining) and immunohistochemical staining of filaggrin of mouse back skin were shown, respectively (magnification: ×400). (c) SCORAD index, scratching score, and dorsal skin thickness were assessed using the criteria described in Section 2. (d) Body weight and spleen weight were measured. Data were expressed as means ± SDs (*n* = 4 per experiment). ^*∗*^ *p* < 0.05 versus DNCB alone. Bar = 250 *μ*m.

To evaluate the effects of VAL on DNCB-induced AD-like skin lesions, we calculated the SCORAD index. When compared to the control group, the DNCB-treated group showed significantly increased physical signs of AD such as erythema, scarring, and edema. However, both low and high doses of VAL significantly decreased the SCORAD index compared with that of the DNCB group (Figures 2(a) and 2(c)). In addition, dorsal skin thickness was significantly decreased by treatment with VAL at a dose of 50 *μ*M (Figure 2(c)). Histological analysis confirmed that VAL inhibited pathological changes including hyperkeratosis and infiltration of inflammatory cells in skin lesions (Figure 2(b)). According to evaluation of filaggrin expression by using immunohistochemistry, filaggrin expression in skin lesion was decreased in DNCB group, while the topical application of VAL resulted in increased expression of filaggrin (Figure 2(b)).

To assess the efficacy of VAL on itching skin, scratching behavior was counted. As shown in Figure 2(c), repeated topical application of DNCB significantly increased scratching behavior, while VAL50 treatment reduced the scratching behavior elicited by DNCB. At the end of the experiment, body weight and spleen weight were measured to assess the general health and immunological status of mice. We found that topical application of VAL did not markedly alter body and spleen weight compared to vehicle-treated mice.

### 3.2. Effect of VAL on Skin, Spleen Cytokines, and Serum IgE Levels in DNCB-Sensitized NC/Nga Mice

**Figure 3:**
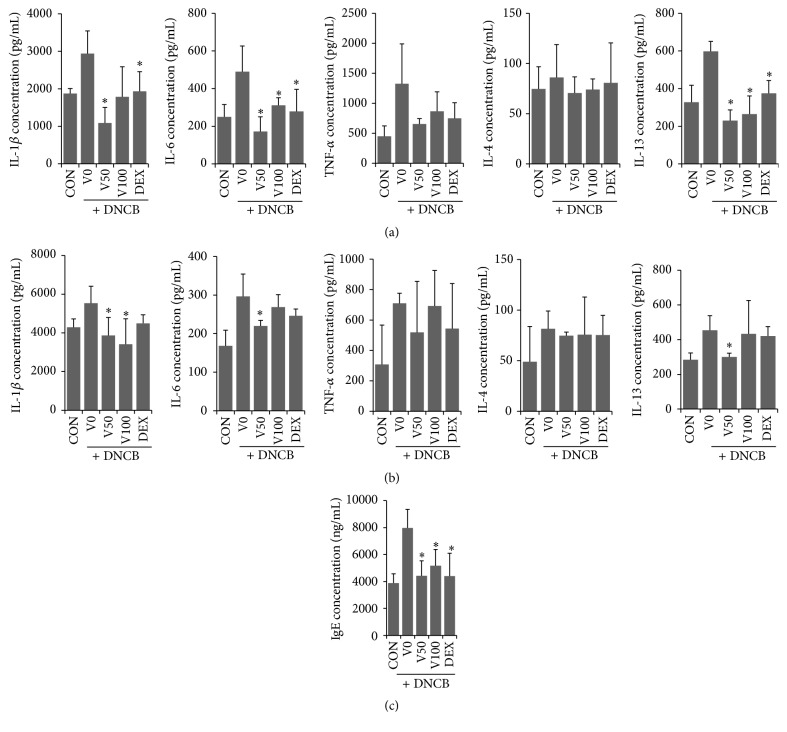
Effects of valencene on DNCB-induced changes in (a, b) proinflammatory cytokines and (c) IgE. (a) Levels of IL-1*β*, IL-4, IL-6, IL-13, and TNF-*α* in (a) skin and (b) splenic tissue were determined by commercial ELISA kits. (c) Serum was isolated from each group of mice after sacrifice, and the serum level of IgE was determined using ELISA. Data were expressed as means ± SDs (*n* = 4 per experiment). ^*∗*^ *p* < 0.05 versus DNCB alone.

To evaluate the effects of VAL on atopic skin inflammation, cytokine levels in skin lesions and spleen tissues were measured. The protein levels of IL-1*β*, IL-6, TNF-*α*, and IL-13 in skin lesions increased in response to DNCB treatment relative to vehicle-treated mice. However, treatment with 50 *μ*M VAL decreased the tissue IL-1*β*, IL-6, and IL-13 levels almost to those observed in response to treatment with DEX, a well-known anti-inflammatory drug (Figure 3(a)). Moreover, VAL treatment markedly suppressed the DNCB-induced increase of IL-1*β*, IL-6, and IL-13 levels in spleen tissue (Figure 3(b)). TNF-*α* levels in skin and spleen tissue were increased by DNCB treatment, but no significant reduction was observed in response to VAL treatment. Next, we assessed the serum level of IgE, which is a mediator in hypersensitivity to environmental allergens. The concentration of IgE in the serum of the DNCB-treated group mice was increased, but this increase was inhibited by VAL in both the low and high dose groups (Figure 3(c)).

### 3.3. Effect of VAL on Proinflammatory Chemokine Expression in TNF-*α*/IFN-*γ*-Stimulated HaCaT Cells

**Figure 4:**
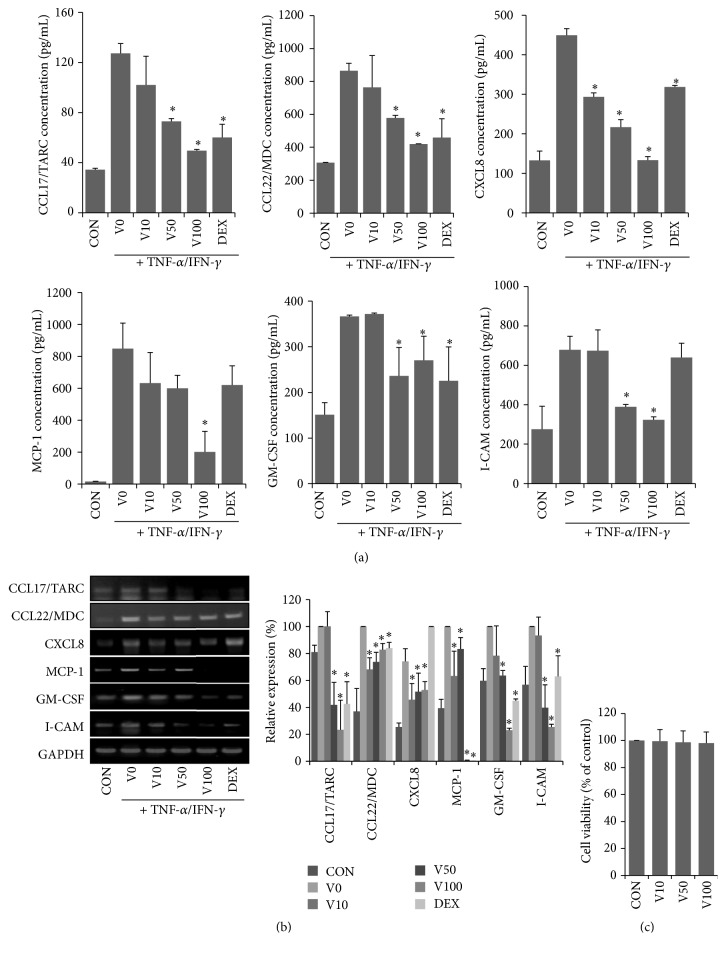
Effects of valencene on the expression/production of chemokines and intercellular adhesion molecule in HaCaT cells. HaCaT keratinocytes were pretreated with valencene (10, 50, or 100 *μ*M; 1 h) and then stimulated with TNF-*α* (10 ng/mL)/IFN-*γ* (10 ng/mL) for 24 h. (a) CCL17/TARC, CCL22/MDC, CXCL8, MCP-1, GM-CSF, and I-CAM levels were determined in culture supernatants using commercial detection kits and (b) corresponding mRNA expression was determined by RT-PCR. (c) Effects of valencene on cell viability was assessed by XTT. HaCaT cells were treated with VAL for 24 h at the indicated concentration. Data were expressed as the means ± SDs (*n* = 3 per experiment). ^*∗*^ *p* < 0.05 versus TNF-*α*/IFN-*γ* alone.

The finding that VAL improved AD-like skin inflammation in NC/Nga mice prompted us to investigate how VAL regulated the development of AD. Mounting evidence suggests that the expression of proinflammatory chemokines in epidermal keratinocytes is associated with atopic skin inflammation. Therefore, we investigated the effects of VAL on TNF-*α*/IFN-*γ*-induced proinflammatory chemokine expression in HaCaT cells. TNF-*α*/IFN-*γ* treatment significantly induced mRNA expression of the AD-related chemokines CCL17/TARC, CCL22/MDC, CXCL8, MCP-1, and GM-CSF, which were significantly inhibited by VAL treatment (Figure 4(b)). Consistent with these results, TNF-*α*/IFN-*γ* treatment significantly provoked the release of chemokines from HaCaT cells. However, VAL treatment significantly inhibited TNF-*α*/IFN-*γ* induced chemokines secretion, and these effects became stronger than those in DEX-treated HaCaT cells at a dose of 100 *μ*M (Figure 4(a)). We also explored the effects of VAL on adhesion molecules. Adhesion molecules play a key role in the recruitment of immunocytes to inflamed skin, and they likely contribute to AD. Pretreatment with VAL significantly inhibited TNF-*α*/IFN-*γ* induced expression/secretion of I-CAM, suggesting that VAL has an anti-inflammatory potential in the epidermis (Figures 4(a) and 4(b)). Since no difference in cell viability was observed using XTT, inhibition of proinflammatory chemokines expression/production was not linked to cytotoxicity (Figure 4(c)).

### 3.4. Effects of VAL on NF-*κ*B p65 and STAT1 Activation in HaCaT Cells

Activation of NF-*κ*B and STAT1 induces the expression of proinflammatory chemokines. Therefore, we focused our analysis on NF-*κ*B p65 and STAT1 translocation in TNF-*α*/IFN-*γ*-treated HaCaT cells. Phosphorylation of I*κ*B-*α* and nuclear translocation of NF-*κ*B p65 and p-STAT1 were highly increased by TNF-*α*/IFN-*γ*-treatment, whereas pretreatment with VAL decreased the levels of p-I*κ*B-*α* and nuclear translocation of NF-*κ*B p65, but not the expression of p-STAT1.

### 3.5. Effect of VAL on Skin Barrier Protein Expression in HaCaT Cells

**Figure 5:**
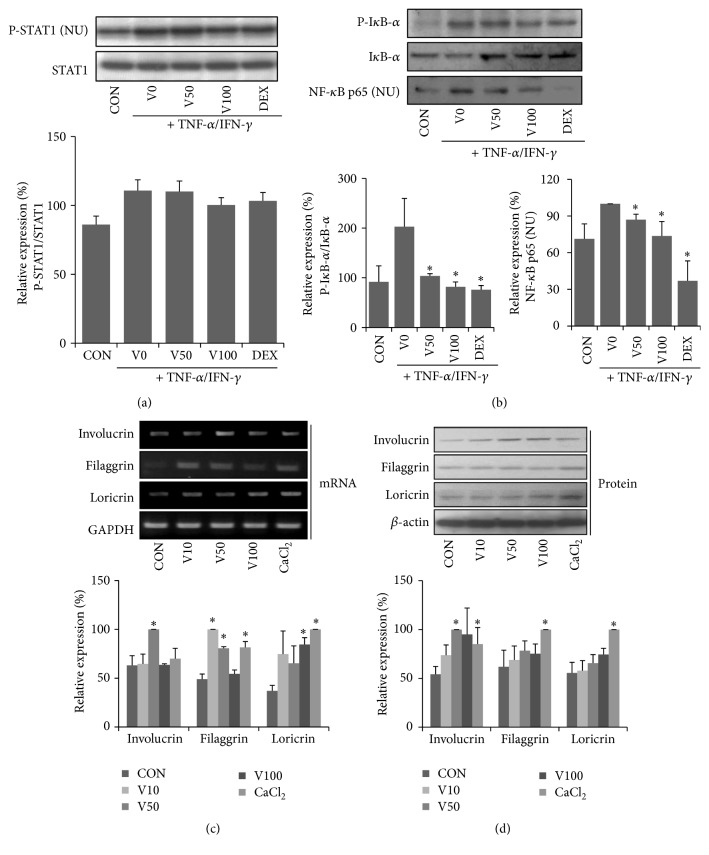
Effects of valencene on activation of the (a) STAT1 and (b) NF-*κ*B signaling pathway in HaCaT cells. HaCaT cells were pretreated with VAL at the indicated concentration for 1 h and then exposed to TNF-*α* (10 ng/mL)/IFN-*γ* (10 ng/mL) for 30 min. Proteins were prepared and analyzed by western blot analysis using specific antibodies against p-I*κ*B-*α*, I*κ*B-*α*, NF-*κ*B p65, STAT1, and p-STAT1. Effects of valencene on involucrin, loricrin, and filaggrin mRNA and protein expression. HaCaT keratinocytes were treated with valencene (10, 50, or 100 *μ*M; 1 h) for 24 hr. (c) mRNA and (d) protein expression were determined by RT-PCR or western blotting, respectively. Data were expressed as the means ± SDs (*n* = 3 per experiment). ^*∗*^ *p* < 0.05 versus TNF-*α*/IFN-*γ* alone or naïve control.

To provide additional evidence for the therapeutic effect of VAL, we investigated its effects on skin barrier protein expression in HaCaT cells. Downregulation of skin barrier proteins such as filaggrin, loricrin, and involucrin increases allergen and pathogen penetration and reduces skin hydration. We exposed HaCaT cells to VAL for 24 h, after which the filaggrin, loricrin, and involucrin mRNA/protein expressions were measured by RT-PCR and western blot analysis. VAL significantly increased involucrin mRNA/protein expressions, and these effects became stronger at a dose of 50 *μ*M (Figures 5(c) and 5(d)).

### 3.6. Effects of VAL on Cytokine Expression/Production in RAW 264.7 Cells

**Figure 6:**
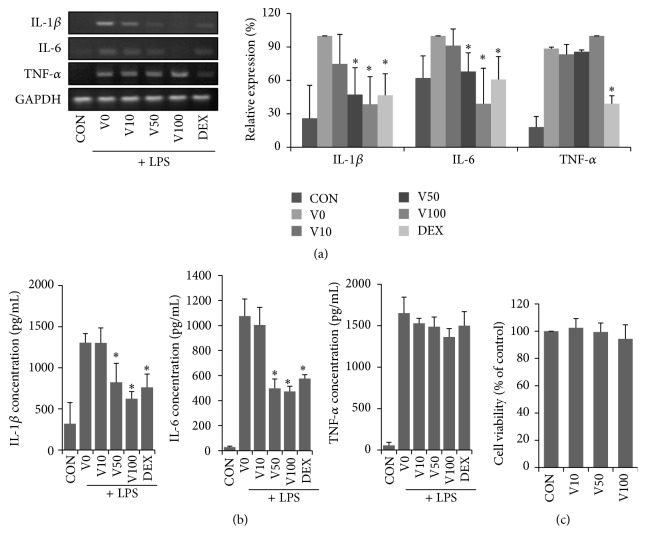
Effects of valencene on the expression/production of proinflammatory cytokines in RAW 264.7 cells. RAW 264.7 macrophages were pretreated with valencene (10, 50, or 100 *μ*M; 1 h) and then stimulated with LPS (1 *μ*g/mL) for 24 h. (a) IL-1*β*, IL-6, and TNF-*α* mRNA levels were determined by RT-PCR and corresponding (b) protein secretion was determined by ELISA. (c) Effects of valencene on cell viability were assessed by XTT. HaCaT cells were treated with VAL for 24 h at the indicated concentration. Data were expressed as means ± SDs (*n* = 3 per experiment). ^*∗*^ *p* < 0.05 versus LPS alone.

Macrophages are known to accumulate in acutely and chronically inflamed skin in AD. Therefore, we confirmed the anti-inflammatory effects of VAL on LPS-treated RAW 264.7 cells, an immortalized macrophage. As shown in Figure 6(a), the levels of IL-1*β*, IL-6, and TNF-*α* mRNA expression were increased by LPS treatment, whereas pretreatment with VAL significantly suppressed IL-1*β* and IL-6 mRNA expression in a nontoxic range (10–100 *μ*M). We also measured secreted IL-1*β*, IL-6, and TNF-*α* protein in the culture supernatant of RAW 264.7 cells. The levels of IL-1*β*, IL-6, and TNF-*α* were highly upregulated in the LPS-treated group when compared with that of the naïve control group. However, VAL treatment decreased the levels of IL-1*β* and IL-6 in a dose-dependent manner. At a dose of 100 *μ*M, IL-1*β* and IL-6 levels were lower than those in the DEX-treated group (Figure 6(c)).

## 4. Discussion

Topical glucocorticoids (GCs) are effective anti-inflammatory drugs frequently used in the treatment of AD [16, 17]. Despite the excellent anti-inflammatory properties of GCs, their undesirable side effects, which include cutaneous atrophy, rebound phenomenon, and decreased skin barrier function, can limit their use [6]. These limited and imperfect therapeutic options have led to substantial public interest in further drug exploration and development for AD. Efforts have been geared toward identifying new phytomedicines and botanical drugs as improved or optimized treatment agents for AD [18]. VAL is one of the essential oils contained in the rhizomes of* C. rotundus* and used to flavor foods and drinks. Several studies have shown that VAL exhibits anti-inflammatory effects. For instance, VAL was effective at preventing and treating cecal ligation and puncture-induced sepsis in mice and significantly inhibited the delayed-type hypersensitivity reaction in picryl chloride induced mice when administered orally at 50–300 mg/kg [13]. In a recent study, VAL inhibited skin photoaging-related ion channels, TRPV1 and ORAI1, and UV-induced melanogenesis in melanoma cells [19]. Therefore, we explored the potential effects of VAL on AD-like skin lesions and investigated the possible underlying mechanism.

We found that VAL possessed potent antiatopic activities, as evidenced by improvement of DNCB-induced AD-like skin lesions in NC/Nga mice. NC/Nga mice have been used as a prominent* in vivo *model for studies of atopic dermatitis pathology and evaluation of potential antiatopic drugs. Similar to a previous report, topical DNCB application led to erythema, edema, itchy skin, and skin-fold thickening with inflammatory immune cell infiltration in skin lesions. Topical application of VAL to mice diminished DNCB-induced skin-fold thickening and improved the SCORAD index, scratching score, and histological lesions.

Because AD is characterized by an impaired skin barrier, we investigated the effects of VAL on expression of skin barrier proteins. Skin barrier provides a physical barrier against mechanical and chemical stresses and prevents the loss of water. An impaired skin barrier can cause many serious complications, such as defective skin hydration, itching, development of dermatitis, and bacterial infections. In AD, a defective skin barrier and an abnormal inflammatory response could become a vicious circle [20]. Skin-resident dendritic cells activated by allergens trigger Th2 polarization and then secrete IL-4, IL-5, and IL-13. These Th2 cytokines increase epidermal thickening, inflammation, and pruritus, while they decrease expression of the barrier proteins [21]. Skin barrier removal by tape stripping has been shown to increase skin levels of thymic stromal lymphopoietin (TSLP), and the resulting Th2 response contributes to the development of AD [22]. In this study, we could observe that VAL inhibited the expression loss of filaggrin, which is one of key structural components of the epidermal barrier and is known to be decreased in AD [23]. These effects might lead to recovery of the barrier function as well as fewer erythematous skin lesions.

Next, we focused on the effects of VAL on proinflammatory cytokines and IgE alterations in NC/Nga mice. In AD, IgE binds to the IgE receptor on mast cells and activates them to secrete proinflammatory and immunomodulatory mediators that cause pruritus in inflammatory skin lesions [24]. Recent evidence suggested that Th2 cytokines could trigger the activation and recruitment of mast cells [25]. Interestingly VAL has demonstrated its antiallergic activity by inhibiting IgE-mediated activation and degranulation of mast cells [14]. In this study, we found that VAL treatment significantly reduced a variety of proinflammatory cytokines, including IL-1*β*, IL-6, and IL-13 in local skin lesions and spleen tissue, and that VAL treatment inhibited serum IgE production. These results indicate that the therapeutic effects of VAL on erythematous and pruritus skin lesions might be mediated, in part by inhibiting proinflammatory cytokines and IgE. These current findings seem theoretically consistent with previous evidence of its antiallergic activity [14]. Moreover, no significant alterations in body and spleen weight were observed in our study, suggesting that topical application of VAL is safe and effective for the treatment of AD.

We further conducted* in vitro* studies to determine the effects of VAL on proinflammatory chemokine secretion in keratinocytes. Keratinocytes are the main cells of the epidermis, the outermost layer of the skin. These cells participate in the pathogenesis of AD by secreting various chemokines. Among these chemokines, TARC/CCL17 and MDC/CCL22 selectively attract Th2 cells, which are predominant in atopic inflammation. CXCL-8, also known as IL-8, amplifies the inflammatory response in AD by recruiting neutrophil into the skin lesions. Serum levels of these chemokines were detected in most cases of atopic dermatitis, and the levels were positively correlated with disease severity in AD patients. Enhanced production of GM-CSF and MCP-1 in keratinocytes may lead to the chronicity of AD lesions by activation of dendritic cells and macrophages. Induction of adhesion molecules in the epidermis is critical to leukocyte adhesion in many inflammatory skin lesions. Skin injury by environmental allergens or scratching activates keratinocytes to release chemokines that induce the expression of adhesion molecules, which may allow lymphocytes to attach and enter the epidermis. I-CAM-1 is constitutively expressed in atopic dermatitis and upregulated by scratching behaviors. Therefore, the effects of VAL on chemokines expression were examined using the HaCaT cell line. VAL suppressed TNF-*α*/IFN-*γ*-induced TARC/CCL17, MDC/CCL22, CCXCL-8, GM-CSF, and ICAM-1 expressions in HaCaT keratinocytes; thus, it may yield therapeutic efficacy by modulating AD-associated chemokines.

Other plant-derived compounds such as saponins and sulforaphane attenuate AD-related chemokine production via the induction of heme oxygenase-1 (HO-1) and blocking NF-*κ*B and STAT1 activation [26, 27]. VAL has been reported to have anti-inflammatory properties through HO-1 induction [13]. However, the molecular mechanism of VAL on NF-*κ*B and STAT1 pathways has not been delineated. Previous reports have shown that NF-*κ*B and STAT1 signaling pathways are involved in the regulation of TARC and MDC mRNA/protein expression in TNF-*α*/IFN-*γ* stimulated HaCaT cells [28]. VAL significantly prevented TNF-a/IFN-*γ*-induced I*κ*-B*α* phosphorylation and NF-*κ*B p65 nuclear translocation in a dose-dependent fashion, but not STAT1 phosphorylation. These results suggest that suppression of NF-*κ*B activation by VAL treatment might leads to decreased production of chemokines in keratinocytes and thus mitigates AD. In addition to its potent anti-inflammatory effects, treatment of keratinocytes with VAL increased mRNA and protein expression of involucrin which is known to provide a cytoskeleton for the cornified envelope [29]. In the case of filaggrin and loricrin, such results were less obvious than in the case of involucrin.

AD research has focused on the regulation of Th2 cells, while little attention has been paid to macrophages. While Th2 cells and their cytokines (e.g., IL-4, IL-5, and IL-13) are increased in acute AD, macrophages, which are known to accumulate in chronically inflamed AD skin lesions, secrete proinflammatory cytokines, resulting in the development of chronic inflammation. Furthermore, macrophages can promote polarization of naïve T cell differentiation toward Th1, which could trigger a complex network of immune responses in chronic AD [30]. We demonstrated that pretreatment with VAL significantly lowered the mRNA and protein levels of IL-1*β* and IL-6 in LPS-stimulated RAW 264.7 macrophages. These results suggest that the prolonged antiatopic effects of VAL in NC/Nga mice over long periods might be mediated in part by inhibition of proinflammatory cytokines secreted by activated macrophages.

## 5. Conclusion

Our results showed that topical application of VAL ameliorates atopic dermatitis symptoms and itching behavior in DNCB-sensitized NC/Nga mice. In studies involving immortalized cell lines, pretreatment with VAL inhibited TNF-*α*/IFN-*γ* induced inflammatory chemokines expression/production in HaCaT keratinocytes through the inhibition of I*κ*B-*α* phosphorylation and nuclear translocation of NF-*κ*B p65. VAL treatment not only modulated the inflammatory response, but also enhanced the expression of the skin barrier protein, involucrin. Pretreatment with VAL inhibited IL-1*β* and IL-6 cytokine secretion in LPS-stimulated RAW 264.7 cell. These results suggest that VAL has a potential therapeutic advantage in the treatment and management of AD.
